# Supplementary material for: Influence of sex, age, and education on mood profile clusters
Source: PLoS One. 2021 Feb 2;16(2):e0245341. doi: 10.1371/journal.pone.0245341 (PMC7853457; doi:10.1371/journal.pone.0245341)
Supplement: Attachment — Submitted filename: PONE-D-20-35037 Response to Reviewers.docx [file pone.0245341.s001.docx]

PONE-D-20-35037

Influence of Sex, Age, and Education on Mood Profile Clusters

Response to Editor

1. We have attempted to ensure that the manuscript now meets PLOS ONE style requirements.
2. The DOI required to access our data will be provided once the paper is accepted for publication.
3. The ORCID for the corresponding author has been validated in Editorial Manager.
4. The ethics statement now appears only in the Methods section of our manuscript.

Response to Reviewer

5. As requested, we have explained the characteristics of the participants in greater detail as they relate to ethnicity, occupation, reason to complete the mood assessment (see lines 96-108).
6. The main contributions of our work have been added at the end of the Discussion (see lines 382-387).
7. Figures have been uploaded to PACE as tif files.

Regards

Dr Peter C Terry
